# Contrasting adaptive strategies to terminal drought-stress gradients in Mediterranean legumes: phenology, productivity, and water relations in wild and domesticated *Lupinus luteus* L.

**DOI:** 10.1093/jxb/eru006

**Published:** 2014-03-03

**Authors:** J. D. Berger, C. Ludwig

**Affiliations:** ^1^CSIRO Plant Industry, Private Bag No. 5, Wembley WA 6913, Australia; ^2^Centre for Legumes in Mediterranean Agriculture, Faculty of Natural and Agricultural Sciences, The University of Western Australia, 35 Stirling Highway, Crawley, WA 6009, Australia

**Keywords:** R- and C-selection, adaptation, crop evolution, terminal drought, water-use and stress onset, phenology, above- and below-ground biomass, productivity.

## Abstract

Our understanding of within-species annual plant adaptation to rainfall gradients is fragmented. Broad-scale ecological applications of Grime’s C-S-R triangle are often superficial, while detailed drought physiology tends to be narrow, focusing on elite cultivars. The former lack the detail to explain how plants respond, while the latter provide little context to investigate trade-offs among traits, to explain where/why these might be adaptive. Ecophysiology, combining the breadth of the former with the detail of the latter, can resolve this disconnect and is applied here to describe adaptive strategies in the Mediterranean legume *Lupinus luteus.* Wild and domesticated material from low- and high-rainfall environments was evaluated under contrasting terminal drought. These opposing environments have selected for contrasting, integrated, adaptive strategies. Long-season, high-rainfall habitats select for competitive (C) traits: delayed phenology, high above- and below-ground biomass, productivity, and fecundity, leading to high water-use and early stress onset. Terminal drought-prone environments select for the opposite: ruderal (R) traits that facilitate drought escape/avoidance but limit reproductive potential. Surprisingly, high-rainfall ecotypes generate lower critical leaf water potentials under water deficit, maintaining higher relative water content than the latter. Given that *L. luteus* evolved in sandy, low-water-holding capacity soils, this represents a bet-hedging response to intermittent self-imposed water-deficits associated with a strongly C-selected adaptive strategy that is therefore redundant in R-selected low-rainfall ecotypes. Domesticated *L. luteus* is even more R-selected, reflecting ongoing selection for early maturity. Introgression of appropriate C-selected adaptive traits from wild germplasm may widen the crop production range.

## Summary

Rainfall gradients select for contrasting, integrated, adaptive strategies in the Mediterranean legume, *Lupinus luteus,* where phenology, productivity, fecundity, and water-use are matched to seasonal rainfall. Profligate high-rainfall ecotypes have developed drought tolerance that is redundant in drought-avoiding low-rainfall ecotypes.

## Introduction

Ecological C-S-R frameworks such as Grime’s triangle (1977) enhance our understanding of plant adaptation by evaluating traits in the context of environmental selection pressure. Widely used to describe species composition and adaptive traits between contrasting environments, they can also provide insight into intra-specific variation, and have been applied in Mediterranean annuals along aridity gradients (Table 1). According to Grime (1977), as rainfall decreases, or becomes more variable (i.e. habitats become more stressful, or likely to be disturbed by terminal drought), reproductive strategies become increasingly conservative (ruderal, R), advancing reproduction and senescence at the expense of biomass production capacity. It is suggested that this limits above- and below-ground resource acquisition (e.g. light, nutrients, water), constraining fitness in terms of yield and fecundity, but allows R-selected plants to escape terminal drought stress. Conversely, with increasing rainfall there is increased selection for competitiveness (C), manifested in delayed phenology, increased biomass production/resource acquisition capacity, and fitness potential (Grime, 1977). However, the Mediterranean studies that align well with Grime’s (1977) predictions tend to be superficial, focusing on traits that are readily measured over large populations (Table 1), emphasizing the role of phenology, biomass, and reproductive effort.

**Table 1.** Intra-specific trait variation in Mediterranean annuals sampled across rainfall gradients

Trait	Low rain	High rain	Species	Germplasm origin	Reference
Phenology	Early	Later	Various (*n*=29)	Syria	Ehrman and Cocks, 1996
	Early	Later	*Biscutella didyma*	Israel	Petrů *et al.*, 2006
	Early	Later	*Triticum dicoccoides*	Israel	Kato *et al.*, 1998
	Early	Later	*Hordeum spontaneum, Avena sterilis*	Israel	Volis, 2007
	Early	Later	*Erucaria hispanica, Brachypodium distachyon, Bromus fasciculatus*	Israel	Aronson *et al.*, 1992
	Early	Later	*Cicer judaicum*	Israel	Ben-David *et al.*, 2010
	Early	Later	*Cicer arietinum*	Mediterranean basin, South Asia, Australia	Berger *et al.*, 2004, 2011
	Early	Later	*Lupinus luteus*	Mediterranean basin	Berger *et al.*, 2008*a*
	Early	Later	*Trifolium glomeratum*	Mediterranean SW Australia	Bennett, 1997
	Early	Later	*Medicago polymorpha*	Italy	Graziano *et al.*, 2010
	Early	Later	*Trifolium subterraneum*	Italy, Mediterranean SW Australia	Nichols *et al.*, 2009; Piano *et al.*, 1996
Biomass	Low	High^*a*^	*T. subterraneum*	Mediterranean SW Australia	Nichols *et al.*, 2009
	Low	High	*T. glomeratum*	Mediterranean SW Australia	Bennett, 1997
	Low	High^*b*^	*C. judaicum*	Israel	Ben-David *et al.*, 2010
	Low	High	*E. hispanica, B. distachyon, B. fasciculatus*	Israel	Aronson *et al.*, 1992
	Low	High	*M. polymorpha*	Italy	Graziano *et al.*, 2010
	Low	High	*Medicago truncatula, M. laciniata*	Tunisia	Yousfi *et al.*, 2010
	Equal	Equal	*L. luteus*	Mediterranean basin	Berger *et al.*, 2008*a*
Reproductive index	High	Low	*E. hispanica, B. distachyon, B. fasciculatus*	Israel	Aronson *et al.*, 1993
	High	Low	*C. arietinum*	Med. basin, South Asia, Australia	Berger *et al.*, 2004
	High	Low	*Biscutella didyma*	Israel	Petrů *et al.*, 2006
	High	Low	*L. luteus*	Mediterranean basin	Berger *et al.*, 2008*a*
	High	Low	*T. subterraneum*	Mediterranean SW Australia	Nichols *et al.*, 2009
Root–shoot ratio	High	Low	*M. truncatula*	Tunisia	Yousfi *et al.*, 2010
	Equal	Equal	*M. laciniata*	Tunisia	Yousfi *et al.*, 2010
	High	Low	*E. hispanica, B. fasciculatus*	Israel	Aronson *et al.*, 1992
Leaf area	Low	High	*Triticum dicoccoides*	Israel	Nevo *et al.*, 1991
	Low	High	*Lupinus albus* (*n*=3)	Portugal, Azores	Rodrigues *et al.*, 1995
Hard seededness	High	Low	*T. subterraneum*	Mediterranean SW Australia	Nichols *et al.*, 2009
	High	Low	Various (*n*=29)	Syria	Ehrman and Cocks, 1996
Seed size	Large	Small	*T. glomeratum*	Mediterranean SW Australia	Bennett, 1997
Growth rates	High	Low	*L. luteus*	Mediterranean basin	Berger *et al.*, 2008*a*
Gas exchange^*c*^	Low	High	*M. truncatula, M. laciniata*	Tunisia	Yousfi *et al.*, 2010
	High	Low	*T. dicoccoides* L.	Israel	Nevo *et al.*, 1991
WUE^*d*^	Low	High	*T. dicoccoides*	Israel	Nevo *et al.*, 1991)
	High	Low	*M. truncatula, M. laciniata*	Tunisia	Yousfi *et al.*, 2010
Water relations^*e*^	High	Low	*M. truncatula, M. laciniata*	Tunisia	Yousfi *et al.*, 2010
	High	Low	*L. albus* (*n*=3)	Portugal, Azores	Rodrigues *et al.*, 1995

^*a*^ Large leaves, broad stems and petioles: large plants at maturity.

^*b*^ Estimated by main stem length.

^*c*^ CO_2_ assimilation (*A*), stomatal conductance (*G*), and transpiration (*T*) per unit leaf area.

^*d*^ WUE: instantaneous water use efficiency (*A*/*T*).

^*e*^ Water relations under deficit: leaf relative water content (RWC), leaf water potential (LWP) (Rodrigues *et al.*, 1995 only), solute concentration and osmotic potential.

By contrast, there is little detailed understanding of adaptive changes in the Mediterranean transition from escape (R-selection) to competition (C-selection) (Table 1). This is unfortunate because the issue of which traits are adaptive where, and at what cost, is much debated among plant scientists, as evidenced by lively argument at the recent Interdrought 4 conference. For example, of the 18 studies listed in Table 1, only two focus on root–shoot ratios or leaf area, and all are very regionally limited. In both studies leaf area increased in the transition from xeric to mesic environments (Nevo *et al.*, 1991; Rodrigues *et al.*, 1995), while root–shoot ratios either decreased, or remained constant (Aronson *et al.*, 1992; Yousfi *et al.*, 2010). Information on plant processes such as growth rates, gas exchange, water relations, and water-use efficiency (WUE) is similarly scarce and sometimes contradictory (Table 1). For example, in xeric *Triticum dicoccoides* rates of CO_2_ assimilation (*A*) and transpiration (*T*) are higher, and instantaneous WUE lower than their mesic counterparts (Nevo *et al.*, 1991), while the opposite was observed in *Medicago truncatula* and *M. laciniata* (Yousfi *et al.*, 2010). The situation is not resolved by widening the scope to temperate climates. North American *Cakile edentula* behaves like *Medicago* (Dudley, 1996), while European *Polygonum arenastrum* resembles *T. dicoccoides* (Geber and Dawson, 1990; Geber and Dawson, 1997). In *Xanthium strumarium*, high gas exchange rates, low WUE, and rapid early growth increased reproductive yield in resource-poor conditions (Lechowicz and Blais, 1988). These observations, augmented by negative correlations between carbon isotope discrimination (δ^13^C; inversely related to WUE: Farquhar *et al.*, 1989), flowering time and yield under drought in a range of crops and model plants (Hall *et al.*, 1990; Craufurd *et al.*, 1991; Ehdaie *et al.*, 1991; McKay *et al.*, 2003), led to the idea that R-selected plants sacrifice WUE in order to maximize growth rates to sustain a short life cycle (Geber and Dawson, 1997).

This contradictory evidence poses a dilemma: does R-selection lead to profligate water use to facilitate a rapid life cycle, while C-selection leads to conservative water use to sustain a longer lifespan, or is it the other way around? An abundance of δ^13^C studies in annual (particularly crop) species does not resolve this issue because δ^13^C is an integrator and not a trait and provides no information on the magnitude of *A* or *T* (Condon *et al.*, 2002). To resolve this dilemma, studies measuring a range of traits, such as stress development and water-use, at a whole plant level are required, using germplasm that has evolved in contrasting environments. Crop wild relatives are an excellent resource for this because they are more diverse than domesticated material, reflect the outcomes of natural selection, are often widely collected and, ideally, include sufficient passport data to characterize the site of collection. This ecophysiological approach, combining the detail of physiology with the breadth of ecological C-S-R theory provides the context to investigate trade-offs among traits in order to explain where and why these might be adaptive.

This approach has been applied here to the Mediterranean legume, *Lupinus luteus* L., comparing wild germplasm from habitats imposing contrasting terminal drought stress with domesticated European and Australian material. This is particularly pertinent in lupin because these recently domesticated crops (~200 years: Hondelmann, 1984) are constrained by limited genetic and adaptive diversity (Berger *et al.*, 2012*a*). By including domesticated material it can be investigated which adaptive strategies were favoured during domestication and what ramifications this has had for the crop. *L. luteus* is endemic to sandy soils in the coastal Mediterranean basin, ranging from low-intermediate to very high annual rainfall (Berger *et al.*, 2008b). It has been shown that wild material from terminal drought-prone habitats flower earlier, produce smaller leaflets, and grow more rapidly than high-rainfall ecotypes (Berger *et al.*, 2008*a*). However, there is no information on traits that define the competitive–ruderal transition, such as fecundity, above- and below-ground biomass production/partitioning, and their effects on water-use and stress development (Table 1). In the present study, contrasting subsets selected from the larger germplasm pool (*n*=100) described in Berger *et al.* (2008*a*, *b*) were evaluated under adequate water supply and terminal drought. There was particular interest in investigating trade-offs between phenology, vegetative and reproductive biomass production, water-use, and stress development among low and high-rainfall ecotypes. In accordance with Grime (1977), it is hypothesized that low-rainfall ecotypes are likely to manifest conservative reproductive strategies that minimize water-use and delay the rate of stress development. Conversely, in high-rainfall ecotypes, competitive traits, such as large leaf area, biomass, and fecundity, leading to profligate water-use were anticipated.

## Materials and methods

Wild *L. luteus* collected from Mediterranean habitats with contrasting terminal drought stress was evaluated alongside domesticated Australian and European germplasm with and without terminal drought stress in a glasshouse pot experiment. To facilitate the labour-intensive measurements in the present study, small germplasm subsets were selected from previous studies (Berger *et al.*, 2008*a*, *b*), clustering along terminal drought-stress gradients (ranked by cluster number: 1=low, 3=high) on the basis of reproductive phase rainfall, mean temperature, and rate of increase (Table 2). Vernalization (28 d at 4 °C prior to planting) and extended photoperiod (16h) was used to minimize phenological variability between genotypes. Plants were sown on 3 June 2008 in 8.1 l pots (ht×diameter: 46×15cm) containing *c*. 12.1kg soil, in five replications arranged in a split plot design with water regime and genotypes as the main- and sub-plots, respectively. Soil was Gingin loam collected on-farm, and mixed with 10% sand to a final water-holding capacity of 22.9% and a pH of 6.5.

**Table 2.** Provenance and collection site seasonal climate of germplasm evaluated in 2008 and 2010 experimentsAbbreviations as follows: Clus, cluster defined in Berger *et al.* (2008*a* ); cv., cultivar; Aus, Australia; Bys, Belarus; Deu, Germany; Esp, Spain; Hun, Hungary; Isr, Israel; Mar, Morocco; Pol, Poland; Prt, Portugal; SUN, former Soviet Union; pre-seas, pre-season; veg, vegetative phase; rep, reproductive phase; temp, temperature.

Cluster category	Habitat	Germplasm origin (*n*)	Cultivar names	Rainfall (mm)		Mean temp (ºC)		Rep temp change	
				Pre-seas	Veg	Rep	Veg	Rep	(ºC d^–1^)
Cluster 1 European cv.	European spring-sown. Low rainfall, cool, rapidly warming veg phase; med rainfall, warm, but cooling rep phase: low terminal drought stress (TDS)	5: Bys, 1; Deu, 1; Hun, 1; Pol, 1; SUN, 1	Grodnenskii, Puissant, Gardenaj, Teo-105 (Wodjil^*b*^), Zhitomirsky 775	306	105	216	10.8	17.2	–0.01
Cluster 3 Australian cv.	Mediterranean short season. Med rainfall, warm veg and rep phases (temp increasing over time): med-high TDS	1: Aus	Pootallong	98	189	97	13.2	14.3	0.10
Cluster 2 wild (W)	Mediterranean long season. High-rainfall cool, frosty veg phase; cool, wet rep phase: low TDS	5: Prt, 4; Esp, 1^*a*^		76	625	468	11.0	14.9	0.07
Cluster 3 wild (W)	(See Cluster 3 above)	5: Isr, 3; Mar,2		14	312	165	15.5	17.3	0.08

^*a*^ Spanish wild germplasm (PNO 22948) only evaluated in 2010.

^*b*^ Wodjil: an Australian cultivar selected from the Polish line Teo-105. (Treated as an Australian, rather than European cultivar in subsequent analyses)

Phenological observations (flowering, podding) were made three times weekly. Terminal drought was established by withholding water 7–10 d after main stem pod set, while the well-watered treatment continued to receive irrigation three times weekly. To minimize evaporative water loss, soil in the terminal drought treatment was covered with white plastic beads. Because there was significant phenological variation between genotypes (Table 3) despite vernalization and photoperiod treatments, it was not possible to apply terminal drought stress synchronously. Accordingly, the terminal drought regime was initiated over four dates starting from 25 August, separated by approximately weekly intervals (see Supplementary Table S1 available at *JXB* online). Despite this, temperatures during the evaluation period (i.e. after the drought treatment was commenced) were remarkably similar across genotypes over time (see Supplementary Table S1 available at *JXB* online). Two late Cluster 2 genotypes evaluated from 16 and 22 September onwards were subject to higher temperatures only after days 30 and 25, respectively, well after the period of peak water-use.

**Table 3.** Phenology, root–shoot ratio, *SLA*, terminal drought water-use, and *RWC* decline of *L. luteus*, categorized by domestication status and habitat of originCluster collection site terminal drought stress intensity increases with cluster number (details in Table 2).

Cluster category	Flowering (d)		Podding (d)		Root–shoot ratio	SLA^*a*^	Water-use (exp. para R)^*b*^		RWC decline (% ºd^–1^)^*c*^	
Year	2008	2010	2008	2010	2010	2010	2008	2010	2008	2010
Cluster 1; European cv.	64	72	75	81	0.33	215.4	0.994	0.989	–0.07	–0.08
Cluster 3; Australian cv.	67	69	78	77		0.993	0.992	–0.05	–0.08	
Cluster 2; wild	87	107	97	114	0.37	172.8	0.985	0.985	–0.21	–0.14
Cluster 3; wild	70	77	82	86	0.30	208.9	0.993	0.992	–0.08	–0.10
Wild contrast: 2 versus 3	<0.001	<0.001	<0.001	<0.001	0.002	<0.001	<0.001	<0.001	<0.001	<0.001
LSD (*P*<0.05)	3	3	4	3	0.04	17.4	0.002	0.004	0.06	0.05

^*a*^ SLA, specific leaf area.

^*b*^ exp. para R, exponential rate of PAW decreases over thermal time since the onset of terminal drought.

^*c*^ % ºd^–1^, linear rate of RWC decrease over thermal time since the onset of terminal drought.

Water relations were described by measuring water-use gravimetrically with an A&D 32kg two decimal place balance, leaf relative water content (RWC) 2–3 times weekly in all treatments, and pre dawn leaf water potential (LWP) in droughted *L. luteus* only. Plant available water (PAW) was expressed as the fraction of transpirable soil water (FTSW) multiplied by 100 (Ritchie, 1981):

(Pot wt day n×final pot wt)/(Initial pot wt−final pot wt)×100

Transpiration rates (*T*) were calculated by dividing water-use quanta by the thermal time elapsed since the last measurement. Relative transpiration rates (*RT*) were calculated to facilitate comparisons that were independent of plant size by dividing *T* at each time point by the maximum *T* measured in that pot. Maximum *T* was estimated by regression, based on the initial linear phase of water-use, when water was freely available, and plants were assumed to be transpiring at maximal rates (Sinclair and Ludlow, 1986).

To measure RWC (Barrs and Weatherley, 1962), leaflets from the most recent fully developed leaf were cut in the early morning (before 9 a.m.), immediately sealed in a pre-weighed glass vial, and kept in shaded conditions. Fresh weights were measured on a four digit Mettler balance within 30min, deionized water added to cover the lower 5mm of the leaf, and the sealed vials left in a well lit area for 8h. Turgid weights were measured after 8h, after removing excess surface water with blotting paper. Dry weights were measured after 24h in a 65 ºC oven. LWP was measured pre-dawn on fully developed young leaves selected and harvested as above (Turner, 1988), wrapped in plastic cling wrap to minimize water loss, and immediately transferred to a Scholander pressure chamber (Series 3000, Soil Moisture Equipment Corp, Santa Bárbara, CA, USA). A dissecting microscope was used to expedite the detection of water at the petiole surface.

Irrigation in the control treatment was ceased after the last genotype in the droughted treatment had matured. Thereafter, above-ground biomass was separated into vegetative and reproductive matter by branch order (main stem, lateral, and basal), and weighed after oven-drying at 60 ºC for 48h. Pods were counted and weighed and used to calculate reproductive index (pod wt/total above-ground biomass).

To test the validity of our work and measure new parameters, the experiment was repeated in 2010, using the methodology outlined above, except that an additional wild Cluster 2 genotype was included (Table 2), and a staggered sowing regime was implemented (10 and 31 May, 14 June) in an attempt to synchronize terminal drought onset. Nevertheless, seven dates of terminal drought initiation were still required, starting from 1 September, separated by approximately 5 d intervals (see Supplementary Table S1 available at *JXB* online). Again evaluation temperatures were remarkably similar across all genotypes over all time periods. In addition, immediately prior to the onset of terminal drought, a destructive harvest was performed to measure above- and below-ground, vegetative and reproductive biomass, the former separated into leaf, stem, and root. Leaf area was also measured destructively at this time.

### Statistical analysis

The data was analysed separately for each year using Genstat V13. Three-way ANOVA was performed with water regime and germplasm provenance (Table 2) as main effects, and genotypes nested within provenance category. In the split-plot ANOVA main plots were nested within blocks. Linear and non-linear regression (*y=A+B(R* ^x^)) was used to analyse plant responses over thermal time to facilitate comparisons between years and between genotypes stressed on different dates. In all analyses, residual plots were generated to identify outliers, and confirm that variance was common and normally distributed. Transformations were made as appropriate.

## Results

### Pre-stress evaluation data

**Fig. 1.**
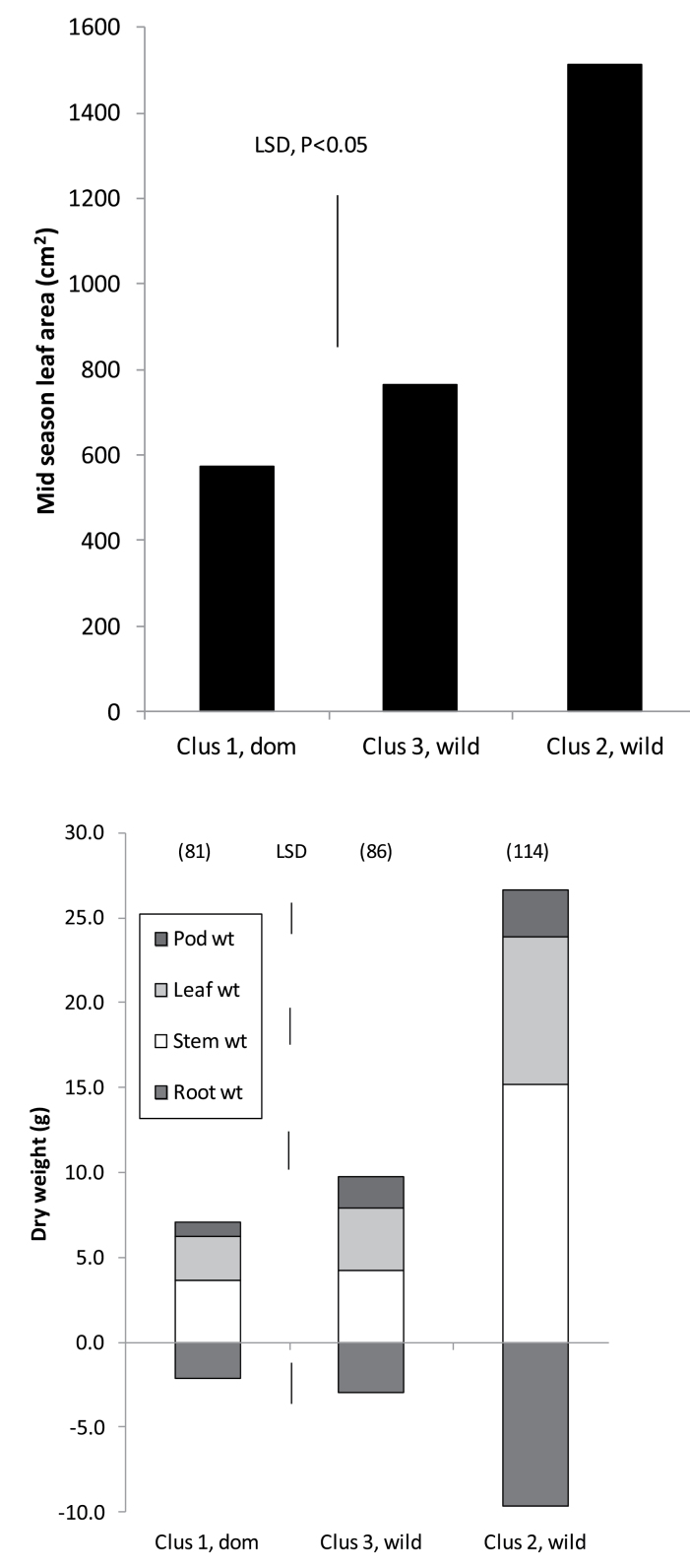
Early reproductive phase leaf area (a) and biomass partitioning (b) in domesticated (Cluster 1) and wild *L. luteus* germplasm collected from contrasting terminal drought-stress habitats (Cluster 2, low; Cluster 3, medium). In (b) root biomass is represented by negative values to emphasize above- and below-ground differences. Values in parentheses are days to first podding; biomass harvests were conducted approximately 7 d later.

Nested ANOVA of genotypes within the provenance category (henceforth referred to as a cluster) highlighted the importance of germplasm origin, with variances generally far exceeding those between genotypes within clusters (although both effects were significant at *P* <0.001). This was particularly evident in plant phenology (Table 3), where domesticated European and Australian cultivars formed flowers and pods consistently earlier than wild low-rainfall ecotypes (Cluster 3) which, in turn, were much earlier than high-rainfall ecotypes (Cluster 2). These phenological differences were reflected in the destructive early reproductive phase biomass measurements made in 2010 (Fig. 1). The late high-rainfall ecotypes accumulated approximately twice the leaf area and mass, and three times the stem and root mass (*P* <0.05) than European cultivars and low-rainfall ecotypes (Fig. 1a, b). Moreover, root–shoot ratios were higher, and specific leaf area lower (eg. thicker leaves in high-rainfall ecotypes (Table 3), while there were no differences among the other two groups. (Note Australian cultivar comparisons were unavailable because of poor germination.)

### Post-stress evaluation: productivity at physiological maturity

**Fig. 2.**
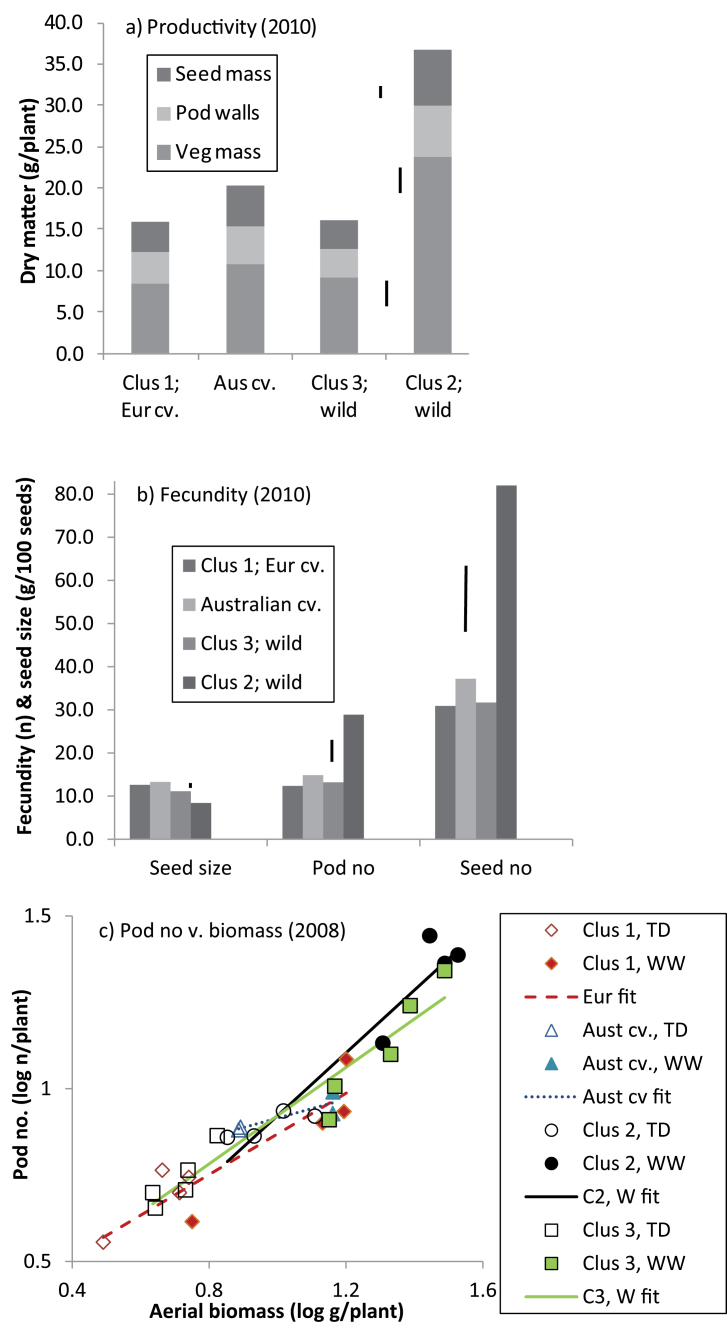
Productivity and fecundity at physiological maturity of *L. luteus* in 2010 (a, b) and 2008 (c). In 2010 (a, b) the main effects are presented because water regime×category interaction was NS. (Error bars represent LSD (*P* <0.05.) The offset LSD in (a) is for pod wt (seed+pod wall). In 2008 (c) the pod no/biomass regression captures 91.5% of variance, with different slopes indicated for clusters (*P* <0.057). (This figure is available in colour at *JXB* online.)

There were also very marked cluster differences for productivity and fecundity at maturity (Fig. 2). Seed, pod, and vegetative weights were 1.6–2.5 times larger in high-rainfall ecotypes than any other group, accounting for all the significant differences (Fig. 2a), while reproductive index was lower (17.7% versus 21.7–22.6%, *P*<0.001). Similarly, high-rainfall ecotypes produced 2.1–2.5 times as many pods and seeds as any other group, again accounting for all significant differences (Fig. 2b). However, seeds from domesticated varieties were larger than wild types (*P* <0.001), while low-rainfall ecotypes produced larger seeds than high-rainfall ecotypes (*P* <0.001). In 2008, highly significant cluster× water regime interactions (*P* <0.001) in the productivity and fecundity data were driven by strong responses to irrigation in reproductive index by high-rainfall ecotypes. Under terminal drought, high-rainfall ecotypes produced almost three times as much vegetative biomass as any other group (*P* <0.001), while pod weights and numbers tended to be similar or lower (data not presented). However, under well-watered conditions, high-rainfall ecotypes were more fecund and productive than any other group in 2008, leading to a steeper rise in pod production as biomass increased (Fig. 2c).

### Water-use and stress onset

**Fig. 3.**
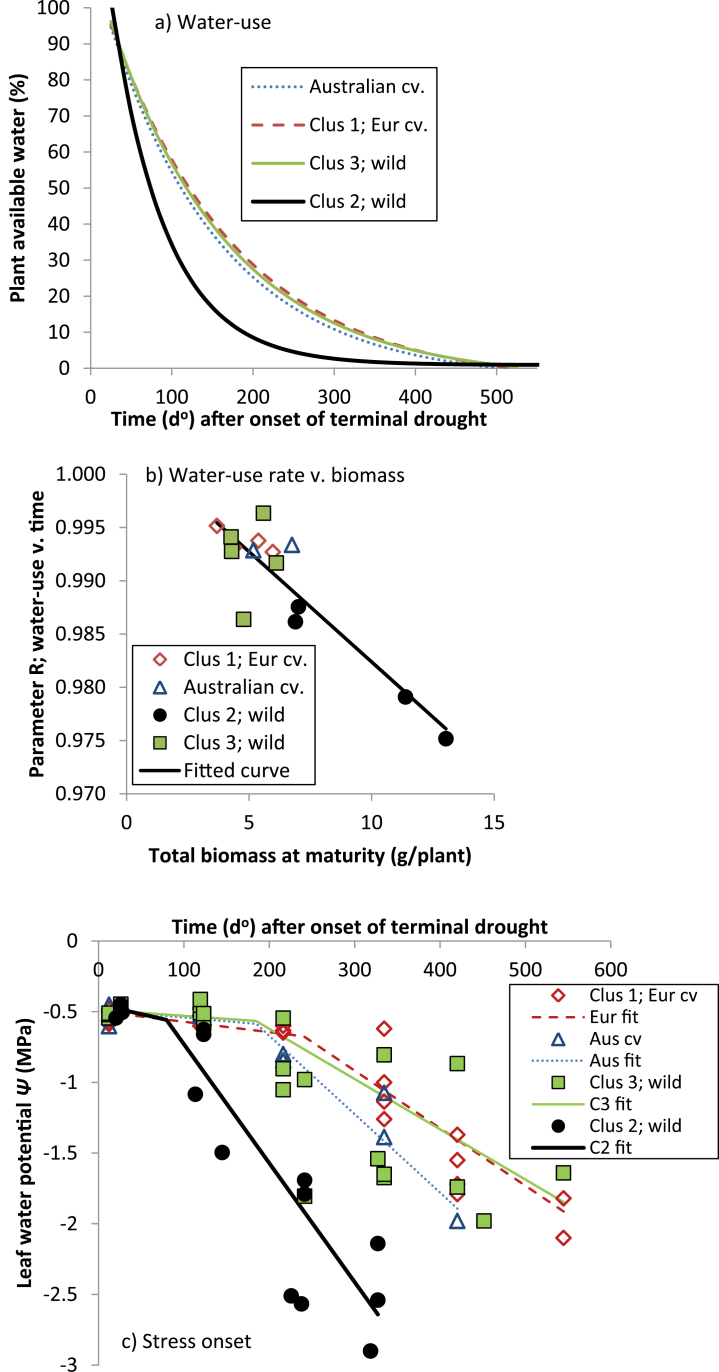
Terminal drought water-use (a, b) and stress onset (c) over time in *L. luteus* in 2008 (2010 data for water-use is similar, and not presented). In (a) exponential curves fitting separate linear and non-linear parameters for clusters explain 91% of variance (93% in 2010). Per cent plant available water (PAW) was calculated using pot weights at field capacity, measured before the onset of terminal drought (1.13 l pot^–1^). In (b) adjusted *r* ^*2*^=0.78 in a linear regression, fitting a common line to all values. In (c) adjusted *r* ^*2*^=0.69 for a broken stick linear regression, fitting separate slopes for provenance categories. (This figure is available in colour at *JXB* online.)

In both 2008 and 2010, regression highlighted differences in water-use and changes in leaf water content (RWC) over time between the two watering regimes (*P* <0.001); slopes remaining flat in the well-watered treatment (water-use: –0.0001–0.0002ml °d^–1^, RWC: –0.002–0.001% °d^–1^), but declining sharply under terminal drought (see below). In both years, terminal drought water-use was best modelled by exponential curves fitting separate parameters for each genotype (variance explained=93.1–96.0%); plant available water (PAW) declining exponentially within 100–300 °Cd (~4–14 d) of withholding irrigation, and then levelling to an asymptote (Fig. 3a). Modelling genotype behaviour by provenance category was almost as effective, capturing 91.2–92.5% of the variance. Water-use was consistently faster in high- than in low-rainfall ecotypes, reflected in differences (*P* <0.001) for *R*, the exponential decline parameter (Table 3), and *A*, the *y* asymptote constant. Water-use rates of domestic *L. luteus* and low-rainfall ecotypes were similar (Fig. 3a). Although rates of water-use were strongly influenced by plant biomass (Fig. 3b), cluster differences remained when transpiration rates were normalized by dividing by aerial biomass at maturity (data not presented). Thus, high-rainfall ecotypes still used water much more quickly than low-rainfall ecotypes, even when accounting for differences in biomass.

In contrast to water-use, changes in RWC were better modelled by linear regression (4-way model: stress thermal time by water regime, by genotypes within provenance categories, accounting for 67.9–71.1% of variance). Well-watered plants retained a high RWC (*c*. 83–90%), while droughted treatments dropped at varying rates to as low as 20%. This contrasting behaviour was well captured by strong interaction between clusters, water regime, and time in both years (*P*=0.003 to *P* <0.001). Under freely-available water, there was no significant decrease in RWC over time in any category (data not presented). In contrast, under terminal drought, the decline in RWC was highest in high-rainfall ecotypes (Table 3), and consistently low in the remaining groups (*P* diff=0.39–0.96).

Leaf water potential (LWP), measured only in droughted *L. luteus* in 2008, was well-modelled by broken stick bi-linear regression, fitting separate curves for the initial flat LWP response to time, and subsequent rapid decline, explaining 79.2% of variance with genotypes nested within clusters. As before, most of this variance was attributed to cluster differences, particularly in the tipping point and subsequent rate of rapid LWP decline (Fig. 3c). Thus the onset of rapid LWP decline occurred much earlier (79.5 °d, 5 d), and proceeded at much higher rates in high-rainfall ecotypes than any other group (*P* diff=0.035 to *P* diff <0.001), accounting for almost all significant slope differences. Accordingly, LWP_C_ in Cluster 2 reached as low as –2.9MPa after 319 °Cd exposure to terminal drought, compared with –1.6 to –2.1MPa after 545 °Cd in the other groups (Fig. 3c).

**Fig. 4.**
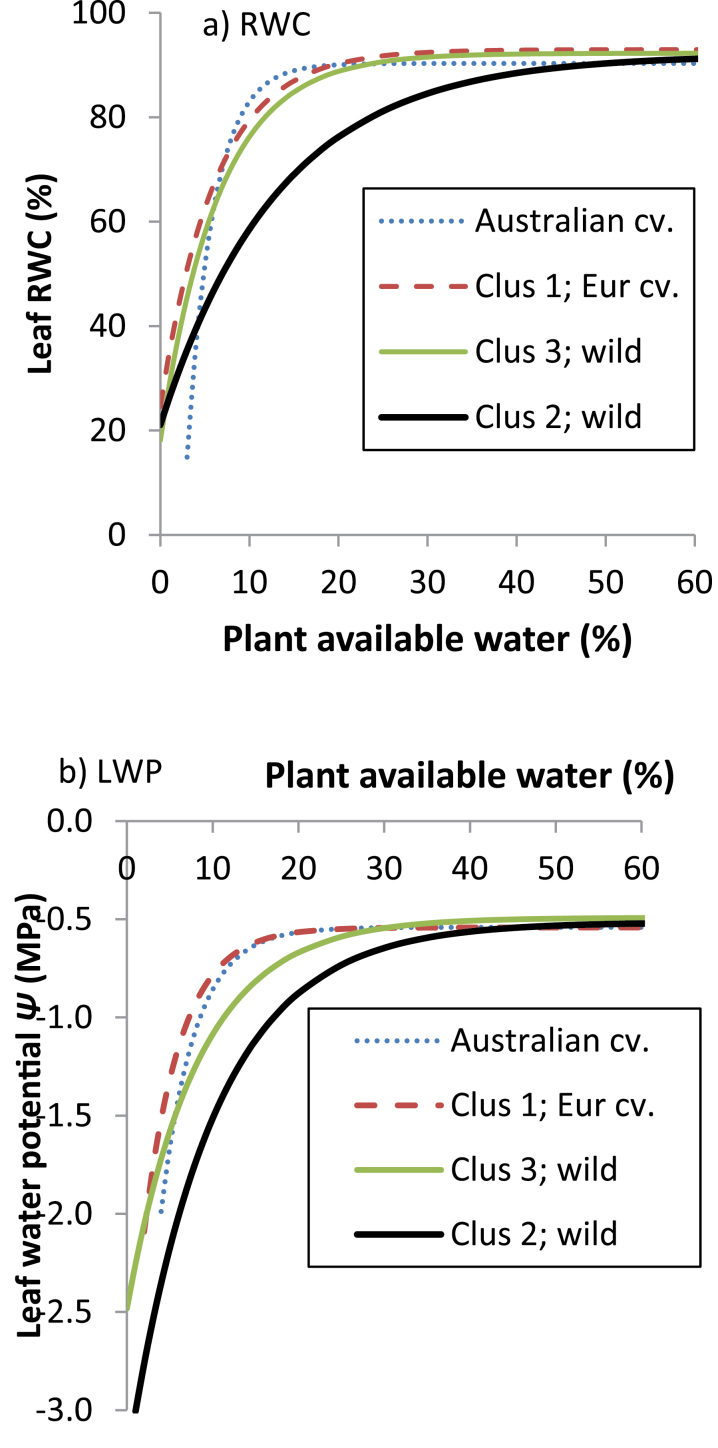
Changes in (a) relative leaf water content (RWC) and (b) leaf water potential (LWP) over diminishingly available water after the onset of terminal drought in 2008 in *L. luteus* (2010 data for RWC is similar, and not presented). Curves represent fitted values from exponential models fitting separate linear and non-linear parameters for clusters (adjusted *r* ^*2*^=0.71–0.78). (This figure is available in colour at *JXB* online.)

RWC and LWP declined exponentially with diminishing PAW (Fig. 4) and, again, most of the variance was explained by cluster rather than genotypic differences within clusters (71.3–78.2%, and 73.5–85.4%, respectively). In both years, the onset of exponential RWC decline occurred earliest in high-rainfall ecotypes, and later (*P*<0.001), at considerably lower PAW in the remaining groups (Fig. 4a). Accordingly, the former have a much shorter asymptotic phase, where RWC is unresponsive to decreasing PAW, than the latter. Similarly, in high-rainfall ecotypes the decrease in LWP occurred much earlier than in its low-rainfall counterparts (Fig. 4b). However, parameter *R* in Australian and European cultivars was much smaller than in both wild groups, reflected in a considerably later onset of LWP decline (Fig. 4b).

**Fig. 5.**
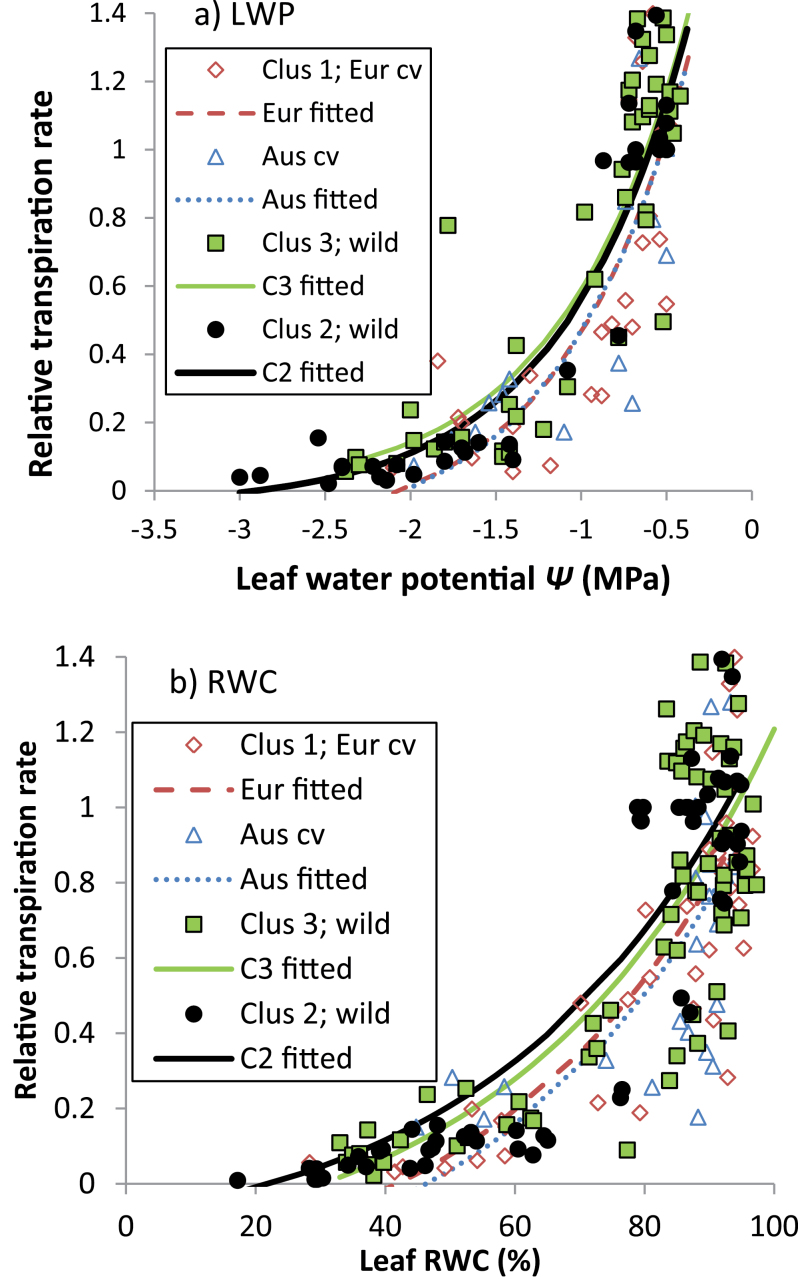
Effect of plant water status (a) LWP and (b) RWC on relative transpiration rate in *L. luteus*. Curves represent fitted values from exponential models fitting separate constant parameters (a) for clusters (adjusted *r* ^*2*^=0.70–0.77). (This figure is available in colour at *JXB* online.)

To examine whether there were differences in the regulation of water-use with increasing stress, relative transpiration rates (*RT*) were regressed against LWP and RWC (Fig. 5a, b). (Because *RT* eliminates leaf area differences, small and large plants, e.g. low- and high-rainfall ecotypes, are compared on an equal basis.) The results were very consistent. *RT* declined with decreasing LWP and RWC at common exponential rates (Fig. 5a, b): there were no genotypic or cluster differences in parameter *R* (*P* diff=0.268–0.996). Conversely, parameter *A*, which determines the *y*-value of the asymptote, did differ between clusters (*P* diff=0.004–0.07) and, therefore, the curves for wild and domesticated material diverged at low LWP and RWC. Consequently, *RT* approached 0 at higher LWP and RWC in domesticated compared with wild material.

**Fig. 6.**
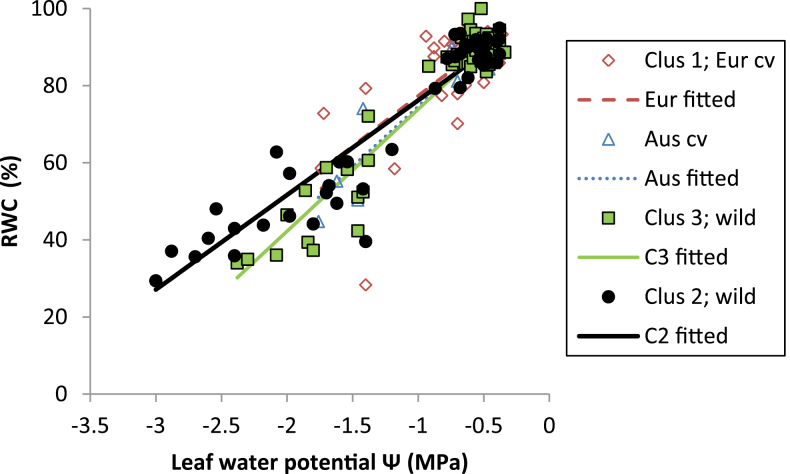
Leaf relative water content declines at different rates over leaf water potential in *L. luteus*, depending on domestication status and habitat of origin. Curves represent fitted values from linear regression, fitting separate intercepts and slopes for clusters (adjusted *r* ^*2*^=0.86). (This figure is available in colour at *JXB* online.)

To investigate plant sensitivity to water deficit stress, RWC was regressed against LWP in a nested linear model (Fig. 6). Slope differences were highly significant (*P*<0.001), and again largely attributed to clusters, rather than genotypes within clusters (accounting for 86.0% and 91.2% of variance, respectively). The decline in RWC over LWP was lower in high- than in low-rainfall ecotypes and Australian cultivars (*P* diff <0.001–0.058). Thus high-rainfall ecotypes were able to maintain RWC under stress better than the other groups, particularly evident at the low final LWP_C_ (Fig. 6).

## Discussion

This research confirms the value of integrated approaches to the study of adaptation to put adaptive traits into context. The use of wild populations that evolved under contrasting terminal drought stress facilitates C-S-R type comparisons, while the inclusion of domesticated material highlights adaptive strategies favoured by breeders, making it possible to speculate how these may have influenced crop development. As outlined below, the results show that Grime’s (1977) C- and R-selected adaptive strategies *do* lead to contrasting water-use and stress onset, leading to rather surprising trade-offs in tolerance to water deficit in *L. luteus*.

In accordance with Grime (1977), *L. luteus* from high rainfall, long-season habitats flowered and set pods considerably later than those from dry, variable-rainfall environments, confirming previous work (Berger *et al.*, 2008*a*), and small, regionally limited studies of *L. angustifolius* (Clements and Cowling, 1994) and *L. albus* (Huyghe, 1997; Simpson, 1986). High below- and above-ground biomass, root–shoot ratios, and leaf area development during the long vegetative phase are likely to provide competitive advantages in the acquisition of growth-limiting resources such as water, nutrients, and light, and be responsible for greater reproductive capacity. Our results suggest that this competitive strategy is always advantageous for high-rainfall ecotypes, assuming adequate water supply at least up to the early reproductive phase. This is remarkably consistent with a small scale (*n*=3) evaluation of wild *L. albus* collected along an Iberian rainfall gradient (Rodrigues *et al.*, 1995), where flowering time and above- and below-ground biomass production in both well-watered conditions and terminal drought was proportional to collection site rainfall. Similar trends were found in Tunisian high- and low-rainfall ecotypes of *M. truncatula* and *M. laciniata* evaluated under a range of water deficits (Yousfi *et al.*, 2010). Like Rodrigues *et al.* (1995), it is shown that these competitive advantages are associated with profligate water-use leading to the early onset of stress in *L. luteus*. High-rainfall ecotypes used most of their PAW within 174 °Cd (11 d), and began the steep linear decline in LWP well before this (79.5 °d, 5 d) (Fig. 3a, c). Indeed, leaf RWC and LWP began to decline at much higher PAW in high- compared with low-rainfall ecotypes (Fig. 4). As stress increased, there was no evidence that high-rainfall ecotypes reduced their consumption compared with low-rainfall ecotypes, as indicated by the common exponential curves in Fig. 5.

Given that *L. luteus* evolved in sandy soils with low-water-holding capacity, the highly competitive high-rainfall adaptive strategy outlined above is risky in a Mediterranean climate. With late phenology, high biomass and high, unregulated water-use, these ecotypes are likely to face repeated water deficits even in a high-rainfall Mediterranean climate as they transpire all PAW. In this context, the ability of high-rainfall ecotypes to generate lower LWP_C_ and maintain higher RWC under extreme stress (Fig. 6) appears to represent a bet-hedging drought-tolerance capacity is redundant in drought-avoiding, R-selected low-rainfall ecotypes. The underlying mechanism is unclear; Old World species such as *L. consentinii* are capable of moderate to high osmoregulation (Gallardo *et al.*, 1994; Turner *et al.*, 1987), while both *L. consentinii* and *L. angustifolius* can double their leaf elasticity under drought (Jensen and Henson, 1990). The extremely limited published data suggests that these adaptive patterns are likely to be species- and habitat-specific along Mediterranean rainfall gradients. For example, *M. truncatula* and *M. laciniata* show the opposite trend: greater osmotic adjustment, maintaining higher leaf RWC under water deficit, in low- compared with high-rainfall ecotypes (Yousfi *et al.*, 2010). Interestingly, these species are found in finer-textured, greater water-holding capacity soils than those favoured by *Lupinus* spp., where the capacity to tolerate water deficits may usefully extend the growing season. Moreover, biomass and transpiration differences between high and low-rainfall *Medicago* were much smaller than observed in *L. luteus*, suggesting that high-rainfall ecotypes were unlikely to self-induce water deficits as a result of profligate water-use.

In contrast to the above, low-rainfall *L. luteus* ecotypes were characterized by a conservative combination of traits where terminal drought is avoided by early phenology, but *also* delayed by lower rates of water-use (associated with reduced biomass and leaf area) and a decreased sensitivity to diminishing water. However, this conservative strategy comes at the cost of reproductive potential, as outlined in the previous discussion. Why then did both European and Australian breeders base their efforts on low-rainfall ecotypes, as suggested by their similarity in the present study? The answer lies in the unique, comparatively recent domestication history of *L. luteus* and *L. angustifolius*. *L. luteus* was introduced to Central Europe (Prussia) as a spring-sown green manure crop in the 19th century as a replacement for *L. albus,* which failed because of its late maturity in the northern summer (Hondelmann, 1984). Hence there was very strong selection for early phenology by early (Gladstones, 1970), and current European breeders (Julier *et al.*, 1995). Subsequently, lupin production shifted to warm, short-season Mediterranean environments in Australia. Australian breeders have selected very strongly for drought escape (Berger *et al.*, 2012*a*), producing very temperature-responsive, early phenology (Berger *et al.*, 2012*b*), augmenting earlier European efforts. This conservative strategy suits Mediterranean water-limited, short season environments by minimizing the probability of drought prematurely terminating the reproductive phase, confirmed by many examples of negative correlations between productivity and phenology (Berger *et al.*, 2004, 2008*a*; Volis, 2007). However, our results suggest that this conservative approach is inappropriate for more productive, longer season environments and excludes potentially adaptive traits for water-limited areas, such as the drought tolerance of high-rainfall ecotypes.

It is concluded that rainfall gradients within the endemic distribution of *L. luteus* have selected for integrated, contrasting adaptive strategies where phenology, biomass accumulation, and partitioning are traded-off against water-use and stress onset. The competitive, profligate high-rainfall ecotypes do not down-regulate water-use under increasing deficit stress any more than those from low-rainfall areas, and appear to have developed a bet-hedging drought tolerance capacity as a result. Conversely, low-rainfall ecotypes have adopted a ruderal adaptive strategy where water-use is minimized, terminal drought avoided, and there is no evidence for drought tolerance. Given that *L. luteus* breeding is based entirely on ruderal, low-rainfall ecotypes, there is improvement potential in introgressing adaptive traits from competitive high-rainfall ecotypes. Having provided a broad context for adaptive strategies in *L. luteus*, future work should focus on the underlying mechanisms. What is the role of phenology: do high-rainfall ecotypes grow biomass faster, or only longer, and how does this impact on water-use, WUE, and competition? How is phenology controlled in contrasting ecotypes; and RWC maintained under low critical LWP? Answering these questions will further our understanding of specific adaptation in *L. luteus* in particular and annual plants in general.

## Supplementary data

Supplementary data can be found at *JXB* online.

Supplementary Table S1. Temperatures (5 d means, °C) recorded during the evaluation of *L. luteus* responses to terminal drought stress.

Supplementary Data
