# Supplementary material for: Contrasting adaptive strategies to terminal drought-stress gradients in Mediterranean legumes: phenology, productivity, and water relations in wild and domesticated Lupinus luteus L
Source: J Exp Bot. 2014 Mar 3;65(21):6219–29. doi: 10.1093/jxb/eru006 (PMC4223984; doi:10.1093/jxb/eru006)
Supplement: Supplementary Data [file supp_65_21_6219__index.html]

Contrasting adaptive strategies to terminal drought-stress gradients in Mediterranean legumes: phenology, productivity, and water relations in wild and domesticated Lupinus luteus L. — Contrasting adaptive strategies to terminal drought-stress gradients in Mediterranean legumes: phenology, productivity, and water relations in wild and domesticated Lupinus luteus L. — Supplementary Data 

# Contrasting adaptive strategies to terminal drought-stress gradients in Mediterranean legumes: phenology, productivity, and water relations in wild and domesticated *Lupinus luteus* L.

## Supplementary Data

Data files

**Files in this Data Supplement:**

- Supplementary Data - Supplementary Data
