# Supplementary material for: Contrasting adaptive strategies to terminal drought-stress gradients in Mediterranean legumes: phenology, productivity, and water relations in wild and domesticated Lupinus luteus L
Source: J Exp Bot. 2014 Mar 3;65(21):6219–29. doi: 10.1093/jxb/eru006 (PMC4223984; doi:10.1093/jxb/eru006)
Supplement: Supplementary Data [file supp_eru006_jexbot110437_file001.pdf]

Supplementary Table 1 (S1): Temperatures (5-day means, C^o) recorded during the evaluation of *L. luteus* responses to terminal drought stress. Despite vernalizing all germplasm, because of variable phenology, terminal drought had to be initiated in a staggered fashion in both 2008 and 2010. Abbreviations as follows: C, cluster defined in Berger et al. (2008b); cv, cultivar; Aus, Australian; Eur, European; W, wild).

Terminal drought start	Genotype (n) & provenance	Day 5	Day 10	Day 15	Day 20	Day 25	Day 30	Mean
25-Aug-08	9 (C1, Eur cv=5; C3, Aus cv=1; C3, wild=3)	13.7	12.9	14.2	15.0	14.3	13.9	14.0
01-Sep-08	4 (C2, wild=2; C3, wild=2)	14.4	13.1	15.3	15.3	14.4	13.7	14.4
16-Sep-08	1 (C2, wild)	15.3	14.4	14.5	14.0	14.9	17.2	15.0
22-Sep-08	1 (C2, wild)	13.6	15.4	13.3	15.7	18.5	21.6	16.3
Min		13.6	12.9	13.3	14.0	14.3	13.7	
Max		15.3	15.4	15.3	15.7	18.5	21.6	
Range		1.8	2.5	1.9	1.7	4.2	7.9	
01-Sep-10	1 (C2, wild)	17.1	17.5	18.2	20.1	18.8	20.0	18.6
03-Sep-10	1 (C3, wild)	17.5	17.6	19.5	18.9	19.6	20.1	18.9
08-Sep-10	4 (C1, Eur cv=1; C2, wild=1; C3, wild=2)	17.6	19.5	18.9	19.6	20.1	19.2	19.1
10-Sep-10	1 (C3, Aus cv)	18.0	20.1	18.8	20.0	19.0	21.5	19.6
14-Sep-10	5 (C1, Eur cv=4; C3, wild=1)	19.7	18.7	20.3	18.7	21.2	20.0	19.8
20-Sep-10	3 (C2, wild=2; C3, wild=1)	18.7	20.3	18.7	21.2	20.0	21.2	20.0
29-Sep-10	1 (C2, wild)	19.9	19.7	20.5	20.7	21.1	22.1	20.7
Min		17.1	17.5	18.2	18.7	18.8	19.2	
Max		19.9	20.3	20.5	21.2	21.2	22.1	
Range		2.8	2.8	2.3	2.5	2.3	2.9	
